# Reduction in Hippocampal Amyloid-β Peptide (Aβ) Content during Glycine-Proline-Glutamate (Gly-Pro-Glu) Co-Administration Is Associated with Changes in Inflammation and Insulin-like Growth Factor (IGF)-I Signaling

**DOI:** 10.3390/ijms25115716

**Published:** 2024-05-24

**Authors:** Laura M. Frago, Emma Burgos-Ramos, María Rodríguez-Pérez, Sandra Canelles, Eduardo Arilla-Ferreiro, Jesús Argente, Manuela G. López, Vicente Barrios

**Affiliations:** 1 Departments of Pediatrics & Pediatric Endocrinology, Hospital Infantil Universitario Niño Jesús, Research Institute “La Princesa”, E-28009 Madrid, Spain; laura.frago@uam.es (L.M.F.); sandra.canelles@salud.madrid.org (S.C.); jesus.argente@uam.es (J.A.); 2 Centro de Investigación Biomédica en Red de Fisiopatología de la Obesidad y Nutriciόn, Instituto de Salud Carlos III, E-28009 Madrid, Spain; 3 Department of Pediatrics, Universidad Autónoma de Madrid, E-28049 Madrid, Spain; 4 Biochemistry Area, Faculty of Environmental Sciences and Biochemistry, Universidad de Castilla-La Mancha, E-45071 Toledo, Spain; emma.burgos@uclm.es (E.B.-R.); maria.rodriguezperez@uclm.es (M.R.-P.); 5 Department of Biological Systems, Faculty of Medicine, Universidad de Alcalá, E-28871 Alcala de Henares, Spain; eduardo.arilla@uah.es; 6 IMDEA, Food Institute, CEIUAM+CSI, Cantoblanco, E-28049 Madrid, Spain; 7 Instituto Teófilo Hernando for Drug Discovery, Department of Pharmacology, Faculty of Medicine, Universidad Autónoma de Madrid, Research Institute “La Princesa”, E-28029 Madrid, Spain; manuela.garcia@uam.es

**Keywords:** Alzheimer’s disease, cytokines, Gly-Pro-Glu, IGF-I signaling, inflammation

## Abstract

Alzheimer’s disease (AD) is characterized by the deposition in the brain of senile plaques composed of amyloid-β peptides (Aβs) that increase inflammation. An endogenous peptide derived from the insulin-like growth factor (IGF)-I, glycine-proline-glutamate (GPE), has IGF-I-sensitizing and neuroprotective actions. Here, we examined the effects of GPE on Aβ levels and hippocampal inflammation generated by the intracerebroventricular infusion of Aβ25-35 for 2 weeks (300 pmol/day) in ovariectomized rats and the signaling-related pathways and levels of Aβ-degrading enzymes associated with these GPE-related effects. GPE prevented the Aβ-induced increase in the phosphorylation of p38 mitogen-activated protein kinase and the reduction in activation of signal transducer and activator of transcription 3, insulin receptor substrate-1, and Akt, as well as on interleukin (IL)-2 and IL-13 levels in the hippocampus. The functionality of somatostatin, measured as the percentage of inhibition of adenylate cyclase activity and the levels of insulin-degrading enzyme, was also preserved by GPE co-treatment. These findings indicate that GPE co-administration may protect from Aβ insult by changing hippocampal cytokine content and somatostatin functionality through regulation of leptin- and IGF-I-signaling pathways that could influence the reduction in Aβ levels through modulation of levels and/or activity of Aβ proteases.

## 1. Introduction

Alzheimer’s disease (AD) is an irreversible pathology that predominantly affects individuals over the age of 65 and is influenced by many factors that contribute to its onset and progression. These include the accumulation of intracellular neurofibrillary tangles and the presence of extracellular deposits of amyloid fibrils at the core of senile plaques, which are associated with neuronal death and a decline in cognitive function [1]. One of the main components of these plaques is amyloid β-peptide (Aβ), produced from the amyloid precursor protein (APP) by sequential enzymatic alternative processing, and it is considered to be a key factor in the pathogenesis of the disease [2].

Inflammation is another important factor contributing to the pathogenesis of AD through the activation of microglia and astrocytes, leading to the secretion of pro-inflammatory cytokines [3]. This dysregulation of interleukins and chemokines in the brain causes neurodegeneration through the modulation of several signaling pathways, most notably nuclear factor kappa B (NFκB) [4]. One of the factors that affect the generation of an inflammatory milieu is the increased production and deposition of Aβ peptides that activate microglia and the subsequent production of cytokines that further enhance Aβ synthesis [5], a vicious circle that leads to neuronal death and pathological changes in astrocytes that impair Aβ clearance [6].

The chronic infusion of Aβ peptides is an experimental approach to AD as it induces hippocampal Aβ deposition associated with neuronal death, deficits in synaptic plasticity and learning [7], and changes in the inflammatory milieu similar to those seen in AD [8,9,10]. In particular, the neurotoxic fragment Aβ25-35 has a more pronounced deleterious effect than Aβ1-42 [11], is associated with the key domain for aggregation, and is also found in the brains of AD patients [12].

Neurodegenerative diseases are a serious health concern worldwide, with a high incidence due to increasing life expectancy and the lack of restorative treatments. Therapies based on the use of different proteins have emerged as a possible strategy due to their high specificity and activity on different biological targets [13]. Several endogenous peptides have anti-apoptotic and neuroprotective properties in the central nervous system, among which glycine-proline-glutamate (GPE), a natural peptide cleaved from the N-terminus of insulin-like growth factor I (IGF)-I, is a protective agent in brain injury [14] and has shown neuroprotective capabilities in experimental models of AD [15,16].

GPE and its analogues have anti-inflammatory properties, which is one of their most important effects, since inflammation favors aggregation processes and decreases the efficiency of glial cells in the processes of the clearance of Aβ aggregates [6]. In this sense, IGF-I is involved in Aβ clearance [17] and also activates the Akt pathway, as does GPE [18]. A decrease in IGF-I sensitivity increases Aβ toxicity, while the activation of its intracellular pathway is associated with an increase in the synthesis and activity of Aβ-degrading enzymes [19], such as insulin-degrading enzyme (IDE).

These aforementioned data suggest that GPE may be useful in AD. However, there is little information on the efficacy of GPE on the possible protective effect against the inflammatory environment generated by the continuous infusion of Aβ25-35 and its relationship with changes in the activation of the Akt pathway. Therefore, we analyzed the activation of some signaling pathways involved in the alterations of the inflammatory environment in the hippocampus of female rats after ovariectomy by studying several pro- and anti-inflammatory cytokines after Aβ infusion in the presence and absence of peripherally administered GPE. We chose the experimental model of female rats after ovariectomy because estrogens reduce Aβ toxicity [20], and most women with AD are elderly and their estrogen levels have already dropped [21]. In addition, the inhibition of estradiol synthesis only affects hippocampal synaptic plasticity in females [22]. As the expression of Aβ-degrading enzymes is related to changes in the Akt pathway, we studied its activation, as well as the leptin signaling that can modulate it and others related to the expression of certain cytokines [23,24]. Finally, since somatostatin (SRIF) modulates the action of Aβ proteases [25], we studied the functionality of this neuropeptide after Aβ infusion and the effect of GPE therapy.

## 2. Results

### 2.1. GPE Reduces Hippocampal Aβ25-35 Levels and the Activation of Inflammatory Pathways after Aβ25-35 Infusion

**Figure 1:**
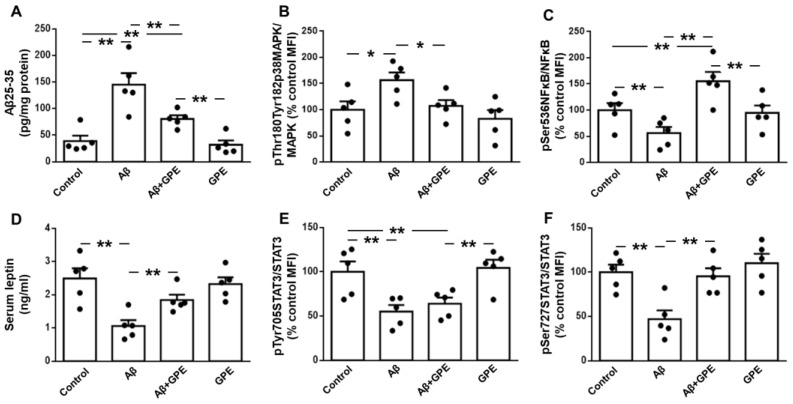
Effects of Aβ25-35 (300 pmol/day) and glycine-proline-glutamate (GPE) co-administration on hippocampal Aβ25-35 levels and phosphorylation of pro-inflammatory and leptin signaling targets. Levels of (**A**) Aβ25-35, relative protein levels of (**B**) p38 mitogen-activated protein kinase (pMAPK) phosphorylated (p) at Thr180 and Tyr182 (pThr180Tyr182p38MAPK) and (**C**) nuclear factor kappa B (NFκB) phosphorylated at Ser536 (pSer536NFkB), (**D**) serum leptin levels and relative protein levels of (**E**) signal transducer and activator of transcription 3 (STAT3) phosphorylated at Tyr705 (pTyr705STAT3), and (**F**) STAT3 phosphorylated at Ser727 (pSer727STAT3) in ovariectomized (Ovx) rats (control), Ovx rats treated with β-amyloid 25-35 peptide (Aβ), Ovx rats treated with Aβ25-35 plus GPE (Aβ + GPE), and Ovx rats treated with GPE (GPE). Data are expressed as mean ± SEM. N = 5. MFI, median fluorescent intensity * *p* < 0.05, ** *p* < 0.01.

Aβ25-35 infusion increased its levels in the hippocampus, and this augmentation was partially blocked by co-administration of GPE. GPE treatment of control rats did not alter Aβ25-35 levels (Figure 1A). We first analyzed activation of p38 mitogen-activated protein kinase (p38MAPK) as Aβ25-35 induces neuronal loss in the rat hippocampus [26]. Phosphorylation of p38MAPK was increased in Aβ25-35-treated rats and GPE prevented the changes induced by Aβ (Figure 1B). Phosphorylation of NFκB at Ser536 is essential for the inhibition of NFκB responses, thereby counteracting inflammatory processes [27]. The infusion of Aβ25-35 reduced NFκB phosphorylation at Ser536 and the co-administration of GPE prevented the changes induced by Aβ25-35, whereas GPE had no effect on NFκB in control rats (Figure 1C). These data suggest that GPE may counteract the activation of these targets.

### 2.2. GPE Partially Counteracts the Inhibitory Effects of Aβ25-35 on the Activation of Leptin Signaling

The chronic infusion of Aβ25-35 reduced serum leptin levels, and the co-administration of GPE prevented the effects of this toxic fragment on circulating leptin concentrations, whereas the administration of GPE to control rats had no effect (Figure 1D). We analyzed the phosphorylation of signal transducer and the activator of transcription 3 (STAT3) at two residues as the phosphorylation of STAT3 at Tyr705 is a prerequisite for its dimerization and nuclear translocation, whereas phosphorylation at Ser 727 is required for DNA binding and transcriptional activation [28]. The phosphorylation of STAT3 at Tyr 705 was reduced in both Aβ25-35- and Aβ25-35-plus-GPE-treated rats (Figure 1E), and the reduction in phosphorylation of STAT3 at Ser 727 induced by Aβ25-35 was avoided by GPE co-administration (Figure 1F). Thus, the Aβ-induced inhibition of leptin signaling is partially prevented by GPE.

### 2.3. Aβ25-35-Induced Downregulation of IGF-I-Related Signaling Is Prevented by GPE Treatment

**Figure 2:**
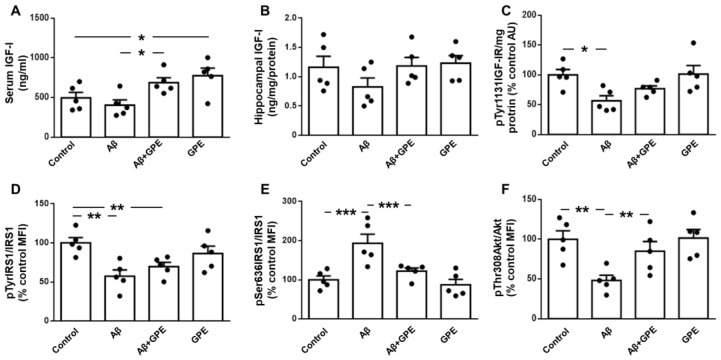
Effects of Aβ25-35 (300 pmol/day) and GPE co-administration on IGF-I levels and IGF-I-related signaling targets. Serum (**A**,**B**) and hippocampal levels of insulin-like growth factor-I (IGF-I) and relative protein levels of (**C**) IGF-I receptor (IGF-IR) phosphorylated at Tyr1131 (pTyr1131IGF-IR), (**D**) insulin receptor substrate 1 (IRS1) phosphorylated at Tyr residues (pTyrIRS1), (**E**) IRS1 phosphorylated at Ser636 (pSer636IRS1), and (**F**) Akt phosphorylated at Thr308 (pThr308Akt) in ovariectomized (Ovx) rats (control), Ovx rats treated with β-amyloid 25-35 peptide (Aβ), Ovx rats treated with Aβ25-35 plus GPE (Aβ + GPE), and Ovx rats treated with GPE (GPE). Data are expressed as mean ± SEM. N = 5. AU, absorbance units, MFI, median fluorescent intensity * *p* < 0.05, ** *p* < 0.01, *** *p* < 0.001.

Serum IGF-I levels were not modified by Aβ25-35 infusion, and GPE increased IGF-I when administered to Aβ25-35 and control rats (Figure 2A). Hippocampal IGF-I levels did not change in any of the experimental groups (Figure 2B). The phosphorylation of the IGF-I receptor (IGF-IR) at specific tyrosine residues is a critical step in the activation of this signaling pathway [29]. The phosphorylation of IGF-IR on Tyr1131 was decreased in Aβ25-35-treated rats, with no changes in the other groups studied (Figure 2C). Phosphorylation of insulin receptor substrate (IRS)-1 on Tyr residues was reduced in Aβ25-35- and Aβ25-35-plus-GPE-treated groups (Figure 2D). The phosphorylation of IRS-1 at the Ser 636 residue inhibits the activation of downstream targets [30]. Aβ25-35 infusion increased phosphorylation at this residue and GPE co-administration prevented this increase (Figure 2E). Finally, Akt phosphorylation at Thr308 was reduced after Aβ25-35 infusion and GPE co-administration prevented this decrease, whereas GPE had no effect on control rats (Figure 2F). These data indicate that Akt signaling is preserved by GPE co-administration.

### 2.4. Effects of Aβ25-35 and GPE on Serum and Hippocampal Cytokine Content

**Figure 3:**
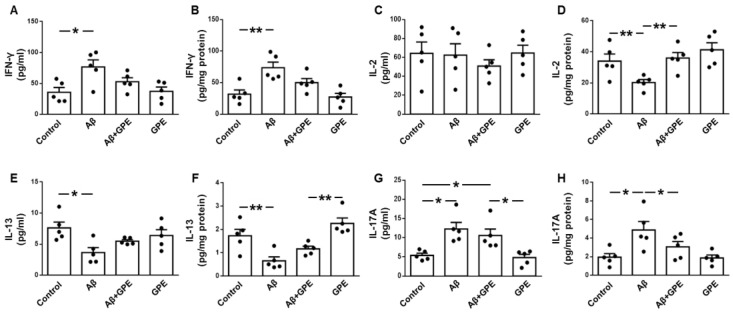
Effects of Aβ25-35 (300 pmol/day) and GPE co-administration on serum and hippocampal cytokine levels. Serum levels of interferon (IFN)-γ (**A**), interleukin (IL)-2 (**C**), IL-13 (**E**), and IL-17A (**G**) and hippocampal concentrations of IFN-γ (**B**), IL-2 (**D**), IL-13 (**F**) and IL-17A (**H**) in ovariectomized (Ovx) rats (control), Ovx rats treated with β-amyloid 25-35 peptide (Aβ), Ovx rats treated with Aβ25-35 plus GPE (Aβ + GPE), and Ovx rats treated with GPE (GPE). Data are expressed as mean ± SEM. N = 5. * *p* < 0.05, ** *p* < 0.01.

The levels of many inflammatory and anti-inflammatory cytokines are altered in AD patients [31]. Circulating levels of interferon-γ (IFN-γ) were increased in Aβ25-35-treated rats (Figure 3A), interleukin (IL)-2 was unchanged (Figure 3C), IL-13 was decreased after Aβ25-35 infusion (Figure 3E), and concentrations of IL-17A were increased in Aβ25-35- and Aβ25-35-plus-GPE-treated rats (Figure 3G). Hippocampal concentrations of IFN-γ were augmented in Aβ25-35-treated rats (Figure 3B), IL-2 levels were reduced after Aβ25-35 infusion and co-administration of GPE- prevented this reduction (Figure 3D), IL-13 levels were reduced after Aβ25-35 infusion, GPE increased IL-13 levels compared to control rats (Figure 3F), IL-17A concentrations were augmented in Aβ25-35-treated rats, and the co-administration of GPE prevented this increase (Figure 3H). These findings show that the inflammatory environment induced by Aβ25-35 can be partially reversed by GPE.

### 2.5. Aβ25-35 and GPE Are Involved in Modulating the Activity of AC and the Levels of an Aβ-Degrading Enzyme

**Figure 4:**
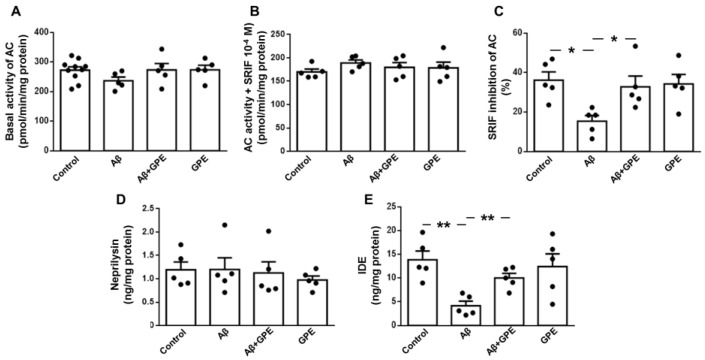
Effects of Aβ25-35 (300 pmol/day) and GPE co-administration on basal adenylyl cyclase (AC) activity (pmol/min/mg protein), as well as on somatostatin (SRIF)-mediated inhibition of AC activity in hippocampal membranes ((**A**) and (**B**), respectively), percentage of SRIF inhibition of AC activity (**C**), levels of neprilysin (**D**) and insulin-degrading enzyme (IDE) (**E**) in ovariectomized (Ovx) rats (control), Ovx rats treated with β-amyloid 25-35 peptide (Aβ), Ovx rats treated with Aβ25-35 plus GPE (Aβ + GPE), and Ovx rats treated with GPE (GPE). Data are expressed as mean ± SEM. N = 5. * *p* < 0.05, ** *p* < 0.01.

SRIF stimulates the activity and levels of Aβ-degrading enzymes [32,33]. We therefore investigated the functionality of this neurotransmitter. As SRIF receptors are coupled to adenylate cyclase (AC) in an inhibitory manner, we examined basal AC activity and the SRIF-mediated inhibition in the membrane fraction from the hippocampus. No differences in basal and inhibited AC activity were observed between the experimental groups (Figure 4A,B, respectively). However, the capacity of SRIF to inhibit basal AC activity was significantly lower in the Aβ25-35-treated group, without changes in the other groups (Figure 4C).

Hippocampal neprilysin levels were similar in all experimental groups (Figure 4D). IDE concentrations were reduced in Aβ25-35-treated rats, and the co-administration of GPE prevented the effects of Aβ infusion. GPE alone had no effect in the control group (Figure 4E). These data suggest that co-administration of GPE is involved in the reduction of hippocampal Aβ25-35 levels through the modulation of IDE and SRIF activity.

### 2.6. Aβ25-35 Content Shows an Inverse Relation to IL-2, SRIF Functionality and IDE

**Figure 5:**
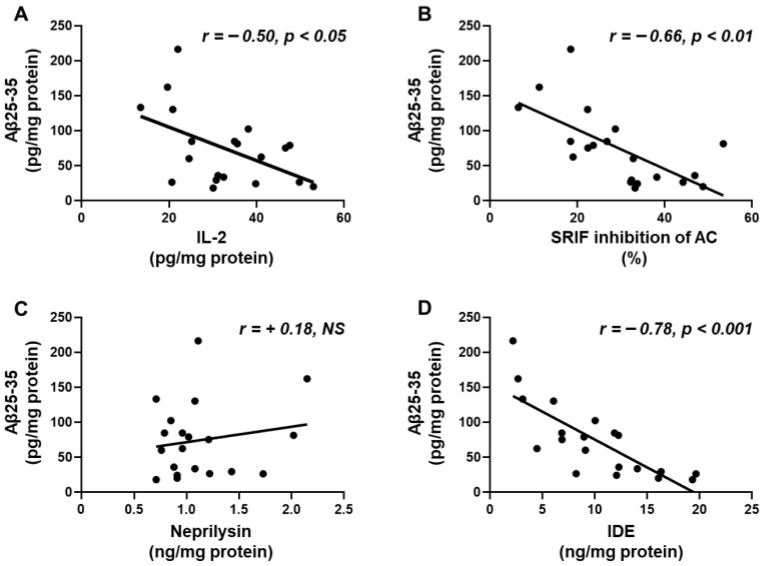
Correlation of Aβ25-35 with (**A**) interleukin (IL)-2 content, (**B**) percentage of inhibition of adenylate cyclase (AC) activity, (**C**) neprilysin, and (**D**) insulin-degrading enzyme (IDE) levels in the hippocampus. Correlation coefficients (r) and *p* values are represented for each analysis. NS, non-significant.

Hippocampal concentrations of Aβ-25-35 were inversely correlated with hippocampal IL-2 content (Figure 5A) and the capacity of SRIF to inhibit AC activity, a measure of SRIF action (Figure 5B). Levels of Aβ25-35 did not show a relationship with neprilysin (Figure 5C) but did show a negative association with IDE (Figure 5D). These correlations may indicate that SRIF activity and IL-2 and IDE levels are involved in the degradation of the toxic fragment.

### 2.7. Correlation of Aβ25-35, SRIF Functionality, and Aβ-Degrading Enzymes with the Phosphorylation of Signaling Targets and Cytokine Levels in the Hippocampus

Linear regression analyses showed a direct correlation of Aβ25-35 levels with the phosphorylation of the pro-inflammatory signaling targets, IFN-γ and IL-17A, and an inverse relationship with phosphorylation of the leptin- and IGF-I-signaling targets, IL-2 and IL-13. In contrast, the percentage inhibition of AC by SRIF and IDE concentrations exhibited an inverse relationship with the phosphorylation of pro-inflammatory targets IFN-γ and IL-17A and a positive correlation with leptin- and IGF-I-intracellular targets IL-2 and IL-13 (Table 1). No correlations were found between neprilysin and the above-mentioned markers. Therefore, changes in intracellular signaling may modulate the factors that modify hippocampal Aβ levels.

### 2.8. GPE Does Not Alter the Aβ25-35-Induced Decrease in Leptin or IGF Signaling in Neuronal Cultures

**Figure 6:**
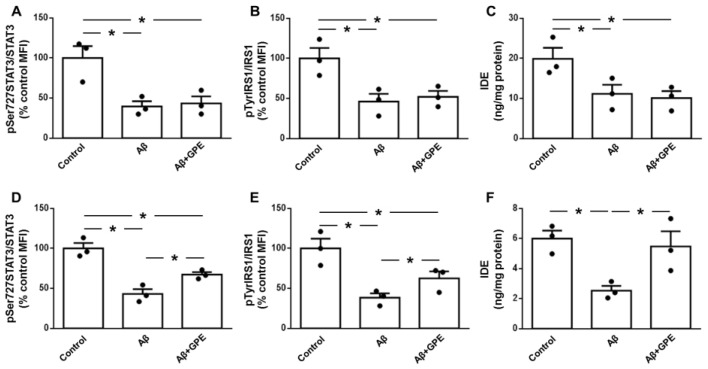
Effects of Aβ25-35 (1 μM) and GPE co-administration on phosphorylation of signaling targets and levels of insulin-degrading enzyme (IDE) in neuronal and glial cultures. Relative protein levels in neuronal and glial cultures of ((**A**) and (**D**), respectively) of the signal transducer and activator of transcription 3 (STAT3) phosphorylated (p) at Ser727 (pSer727STAT3), ((**B**) and (**E**), respectively) insulin receptor substrate 1 (IRS1) phosphorylated at Tyr residues (pTyrIRS1) and protein concentrations, and ((**C**) and (**F**), respectively) insulin-degrading enzyme (IDE). Data are expressed as mean ± SEM. N = 5. * *p* < 0.05.

The addition of Aβ25-35 to neuronal cultures reduced phosphorylation of STAT3 on Ser727 and IRS1 at Tyr residues (Figure 6A,B, respectively), as well as the IDE content in these cultures (Figure 6C). GPE-coadministration had no effect on Aβ25-35-induced changes (Figure 6A–C). These data suggest that GPE does not appear to act directly on neuronal populations.

### 2.9. GPE Co-Administration Modifies Aβ25-35-Induced Changes in Glial Cell Signaling and Cytokine Secretion

Aβ25-35 decreased the phosphorylation of STAT3 and IRS1 and IDE levels in glial cells (Figure 6D–F, respectively). The co-administration of GPE partially prevented changes in STAT3 and IRS1 phosphorylation (Figure 6D–E) and fully prevented Aβ25-35-induced IDE reduction (Figure 6F).

**Figure 7:**
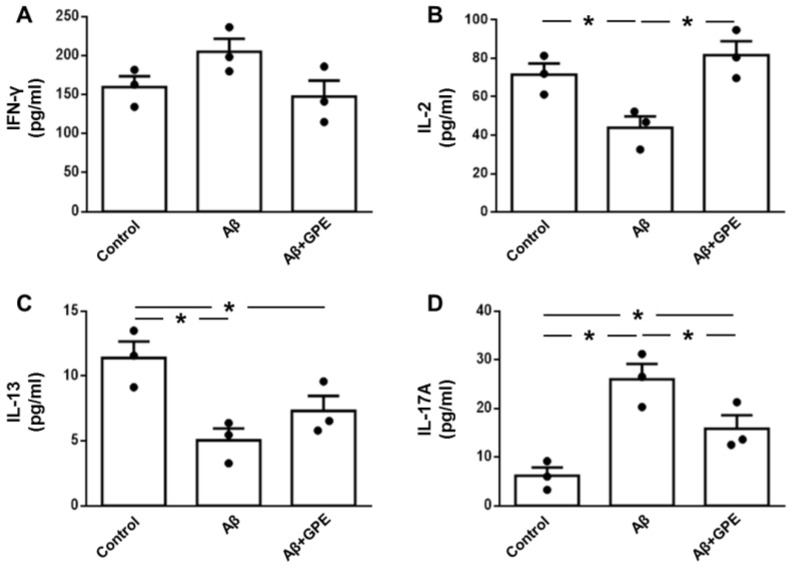
Effects of Aβ25-35 (1 μM) and GPE co-administration on interleukin secretion in glial cultures. Protein levels in culture media of interferon (IFN)-γ (**A**), interleukin (IL)-2 (**B**), IL-13 (**C**), and IL-17A (**D**). Data are expressed as mean ± SEM. N = 5. * *p* < 0.05.

Levels of IFN-γ in culture media were unaffected by Aβ-25-35 or GPE co-administration (Figure 7A). Aβ25-35 induced a reduction in IL-2 levels that was prevented by the co-administration of GPE (Figure 7B). Concentrations of IL-13 were reduced after the addition of Aβ25-35, and GPE had no effect (Figure 7C). Aβ-25-35 augmented IL-17A levels, and GPE co-administration partially prevented these changes (Figure 7D).

## 3. Discussion

### 3.1. Summary

Extracellular plaque-like deposits within the hippocampus lead to cognitive impairment and cause inflammation as Aβ protofibrils activate microglia, triggering an inflammatory response and the release of neurotoxic cytokines [34]. This study was designed to analyze the effect of a neuroprotective agent derived from IGF-I, the tripeptide GPE, on the changes in the inflammatory environment of the hippocampus and its possible relationship with the activation of various signaling pathways related to these processes. Here, we report that GPE blocks most of the changes in cytokine content in the hippocampus induced by the continuous infusion of Aβ and that this effect may be mediated by preserving the activation of leptin- and IGF-I-related signaling pathways. In addition, we show that the decrease in IDE after Aβ insult is blocked by the co-administration of GPE, contributing to the reduction in hippocampal Aβ levels.

### 3.2. Aβ-Induced Inflammation and GPE Effects on Signaling and Cytokine Environment

Our data show an increase in the activation of pro-inflammatory signaling targets after Aβ infusion. There was an augmentation in p38MAPK phosphorylation and a reduction in the Ser residue of NFκB, which activates this molecule. As we have found in this study, it has previously been reported that the activation of these targets increases the levels of IFN-γ and IL-17A [35] while decreasing the content of the anti-inflammatory IL-13 [36]. A striking finding was the decrease in hippocampal levels of IL-2, a cytokine classically associated with an inflammatory profile. This finding may be related to the decrease in STAT-3 activation, since the phosphorylation of this target increases the levels of this interleukin and its subsequent signaling [37,38]. One of the mechanisms that may influence STAT-3 phosphorylation is Aβ itself, as it is a negative allosteric modulator of the leptin receptor [39], with consequent decreased activation of downstream targets.

The co-administration of GPE was able to modify most of the Aβ-induced changes in signaling pathways and inflammation. Hence, the systemic administration of GPE reduces p38MAPK activation [40] and suppresses the NFκB inflammatory pathway in experimental models of neurodegenerative disease [41]. The effects of GPE mimic those exerted by IGF-I, increasing Akt activation [16], although it does not bind to IGF-IR. The activation of the Akt pathway may be favored by the increase in leptin signaling after GPE co-administration, as was reported in other situations [42], and the increase in serum leptin levels may explain, at least in part, the activation in its signaling and subsequent phosphorylation of IGF-I-related targets. In this way, the disruption of leptin signaling in a mouse model of AD reduces Akt in parallel with the upregulation of the suppressor of cytokine signaling 3 (SOCS3) in the hippocampus [43], and we have demonstrated that the central infusion of leptin reduced the association of SOCS3 with IGF-IR, increasing its phosphorylation and activation of downstream targets [44].

A role for reactive glia in neuronal damage and recovery has been reported. Treatment with GPE suppresses microglial proliferation and prevents the loss of astrocytes after injury [45]. Our “in vitro” experiments seem to demonstrate that glial cells are involved in the modifications of cytokine levels in the hippocampus, after both Aβ administration and co-treatment with GPE, which partially or totally restores the levels of cytokines affected by Aβ infusion. Aβ activates astrocytes, inducing an increase in GFAP, vimentin, and pro-inflammatory cytokines, whereas GPE normalizes the GFAP, vimentin, and cytokine profile [46,47]. Hence, GPE binds to astrocytes and reduces brain inflammation [48].

Cytokines can also regulate signaling themselves. Thus, IL-2 synergizes with IGF-I in processes related to memory enhancement in experimental animals and promotes Akt activation in homeostatic processes of proliferation [49]. IL-13 also has anti-apoptotic and proliferative effects in different tissues modulating the pathways analyzed here. The antiapoptotic effects of this interleukin have been described through the activation of the Akt pathway [50] and proliferative effects by increasing STAT-3 phosphorylation [51]. Therefore, among the multiple activities associated with the pathological conditions of AD [52], we may speculate that GPE may be prevent/reverse Aβ damage through changes in interrelated signaling pathways and cytokine profiles, thereby enhancing its beneficial actions on this disease.

### 3.3. SRIF Functionality and Aβ-Degrading Enzymes

This study shows that the deleterious effects of Aβ on SRIF functionality are blocked by GPE. Although the regulatory mechanisms of SRIF tone are partially unknown, both our previous results [13] and the new data included in this study suggest that the activation of leptin and IGF-I signaling may be involved in the protective effect of GPE on this neurotransmitter. Leptin may be involved in the preservation of SRIF cells against Aβ effects as this adipokine protects against Aβ-induced cell death through a STAT3-dependent mechanism [53]. IGF-I-related signaling may promote SRIF synthesis, as Akt activation promotes CREB phosphorylation, which induces the expression of SRIF and its receptors [54].

One of the most striking findings was the reduction in Aβ levels when GPE was co-administered. In this way, the increase in SRIFergic tone may modulate the expression of Aβ-degrading proteases [33]. Here, we found an increase in hippocampal IDE levels, with no differences in neprilysin content. Nevertheless, as the activity of neprilysin is regulated by SRIF [55], the increased functionality of this neuropeptide suggests an active role of this protease in the decrease in Aβ levels. IDE may also be regulated by phosphatidylinositol 3-kinase (PI3K) activation, as factors that augment Akt phosphorylation may raise IDE expression and synthesis [56].

### 3.4. Regulation of Aβ Levels by Other Factors

We cannot rule out additional factors mediating the effects of GPE on Aβ levels. This tripeptide can be metabolized to cycloprolylglycine, another important metabolite of IGF-I [57]. This dipeptide improves memory and reduces the Aβ plaque load in double transgenic mice APP/presenilin-1 (PS1) [58]. Leptin signaling may also be involved in the depletion of Aβ in the hippocampus, as has been reported in diabetic rats subjected to high-intensity interval training, which showed an increase in leptin receptor, Janus kinase 2 (JAK2), and STAT3 and a concomitant reduction in glycogen synthase kinase 3β, neurofibrillary tangles, and Aβ levels [59].

Several interleukins may also be involved in the decrease in Aβ content, particularly IL-2 and IL-13, which increase after the co-administration of GPE. For example, a decrease in IL-2 levels has been found in hippocampal biopsies from patients with AD. Furthermore, in the hippocampus of APP/PS1 transgenic mice, IL-2 administration induces the activation and regrouping of astrocytes around amyloid plaques, decreases Aβ content, and improves synaptic plasticity [60]. The central infusion of IL-13 ameliorated cognitive deficits via degradation and clearance of intra- and extraneuronal Aβ peptides in APP23 mice by modulating Aβ-degrading proteases [61]. The decrease in the hippocampal content of IL-17A levels after GPE co-treatment may also be related to diminished Aβ levels. Thus, this interleukin promotes AD progression in the APP/PS1 mouse model by increasing neuroinflammation through the NFκB pathway and Aβ deposition [62].

### 3.5. Limitations of the Study

Clearly, more research is needed to better understand the role of changes in the activation of signaling pathways and their relationship with inflammatory markers in experimental models of this disease. Further “in vitro” studies could provide additional information on the effects of these cytokines in relation to changes in the activation of these signaling targets and enzymes involved in Aβ degradation, as well as the cell populations involved in these actions. Another aspect to take into account is the lack of memory testing in this study and the relationship with changes in peripheral inflammation. Our results showed inflammatory changes in the circulation, although they were more pronounced in the hippocampus. Some studies have shown an association between the increase in serum cytokines and the progressive decline in spatial memory after Aβ infusion [63]. In relation to this finding, there are also reports showing the association between biomarkers of inflammation and the degree of dementia in AD patients [64].

## 4. Materials and Methods

### 4.1. Materials

All chemicals were purchased from Merck (Darmstadt, Germany) unless otherwise noted. Osmotic minipumps were from Alzet (Palo Alto, CA, USA).

### 4.2. Preparation of Aβ25-35

Aβ25-35 peptide was prepared according to the method reported by Pike et al. [65]. This peptide was dissolved in 1% acetic acid according to the manufacturer’s instructions and aged prior to administration by incubation at 37 °C for 4 days to induce aggregation. One day before the implantation, osmotic minipumps were connected and filled with 200 μL of Aβ25-35 solution and primed in 0.9% saline solution at 37 °C overnight [66].

### 4.3. Animals and Experimental Design

This study was approved by the Ethics Committee of the Universidad de Alcalá de Henares (SAF 2010–22277, Ministerio de Ciencia y Tecnología) and complied with Royal Decree 1201/2005 (Boletín Oficial del Estado, BOE No. 252) pertaining to the protection of experimental animals and with the European Communities Council Directive (86/609/EEC). Female Wistar rats, weighing 250–280 g, supplied by Harlan Laboratories Models S.L. (Barcelona, Spain), were housed in groups of 2 rats per cage on a 12 h light/dark cycle with free access to water and food and were allowed one week of acclimatization before the start of the experiments. Care was taken to use the minimum number of animals.

Twenty female Wistar rats of 8 weeks of age were bilaterally ovariectomized under anesthesia (0.02 mL of ketamine/100 g body weight and 0.04 mL of xylazine/100 g body weight) as previously reported [47]. Three weeks after ovariectomy, the animals were distributed into four groups. In the first group, a cannula attached to an osmotic minipump was implanted in the right cerebral ventricle (−0.3 mm anteroposterior, 1.1 mm lateral) and Aβ25-35 was infused for 14 days (300 pmol/day, infusion rate 0.5 μL/h) as described [67]. In a second group, Aβ25-35 was infused at the same time and dose, and three intraperitoneal injections of GPE (300 μg, dissolved in isotonic saline) were administered at 0, 6, and 12 days. Another group received GPE alone, as described for the previous group. Control rats received vehicles by the same administration routes. On day 14, the rats were sacrificed, the serum was stored at −80 °C, and the brain was dissected on ice to obtain the hippocampus [68].

### 4.4. Tissue Homogenization and Protein Quantification

For immunodetection of Aβ25-35, phosphorylated (p) Thr308Akt, Akt, IDE, IFN-γ, IGF-I, IGF-IR, IL-2, IL-13, IL-17A, pSer636-IRS1, pTyr-IRS1, IRS1, pThr180/Tyr182-p38MAPK, p38MAPK, neprilysin, pSer536-NFkB, NFκB, pSer727STAT3, pTyr705STAT3 and STAT3, and hippocampus was homogenized on ice in 400 μL of lysis buffer (Merck). Lysates were frozen for 12 h at −80 °C and then centrifuged at 12,000× *g* for 5 min at 4 °C. Supernatants were stored at −80 °C until assayed. Protein levels were determined by the Bradford method (Bio-Rad Laboratories, Madrid, Spain).

### 4.5. ELISAs

#### 4.5.1. Aβ25-35

Hippocampal levels of Aβ25-35 were determined using an ELISA kit from Blue Gene Biotech (Shanghai, China), with a monoclonal capture antibody against Aβ25-35 and another detection antibody conjugated to horseradish peroxidase (HRP). After 60 min incubation at 37 °C, the wells were washed and incubated with a substrate and the absorbance was read at 450 nm.

#### 4.5.2. Aβ-Degrading Enzymes

Neprilysin levels in the hippocampus were measured using an ELISA from Cusabio (Wuhan, China). Homogenates were incubated with a capture antibody for 120 min at 37 °C. Once samples were removed, a biotin antibody was added. After 60 min, HRP-avidin and a substrate were added until the color developed.

Levels of IDE were assessed using a kit from Cloud-Clone Corp. (Houston, TX, USA). After incubating the homogenates for 120 min with a biotin conjugated-IDE antibody, an avidin-HRP complex was added, incubated for 90 min at 37 °C, and subsequently washed. The substrate solution was added until a blue color developed.

#### 4.5.3. IGF-I

Serum and hippocampal IGF-I concentrations were analyzed using an ELISA kit from R&D Systems (Minneapolis, MN, USA). Serum and homogenates were incubated with a monoclonal anti-IGF-I capture antibody for 120 min at 25 °C. After washing, conjugate was added and incubated for 120 min. Wells were washed again and incubated with a substrate solution for 30 min, and the absorbance was read at 450 nm.

#### 4.5.4. Phosphorylation of IGF-I Receptor

The assay (Cell Signaling Technology, Danvers, MA, USA) detects levels of IGF-I receptor protein when phosphorylated at Tyr1131 residue. Homogenates were incubated for 120 min at 37 °C in a plate coated with the pTyr1131-IGF-I antibody. After washing, a detection antibody was added and incubated at 37 °C for 60 min. Afterwards, the plate was washed again, and an HRP-linked secondary antibody was added and incubated 37 °C for 30 min. After washing, the substrate was added, and the absorbance was read at 450 nm.

#### 4.5.5. Leptin

Serum leptin levels were measured using a kit from Merck. Standards, controls, and samples were added together with a capture antibody, to a plate coated with a capture antibody. After 120 min of incubation, the plate was washed, and the enzyme was added and incubated for 30 min. After washing, the substrate was added until the development of a blue color and then read at 450 nm.

The intra- and inter-assay coefficients of variation were lower than 10% for all assays.

### 4.6. Multiplexed Bead Immunoassays

Phosphorylated and total levels of Akt, IRS1, p38MAPK, NFκB, and STAT3 in the hippocampus, as well as concentrations of IFN-γ, IL-2, IL-13, and IL-17A in the serum and hippocampus, were measured using multiplexed bead immunoassays (Bio-Rad Laboratories and Merck) following the manufacturer’s recommendations. Beads conjugated to antibodies and serum or homogenates (25 μL each) were incubated, and antibody conjugated to biotin was added and incubated. Then, beads were incubated with streptavidin-phycoerythrin. At least 50 beads per variable were examined in the Bio-Plex suspension array system 200 (Bio-Rad Laboratories). Raw data (median fluorescence intensity, MFI) were evaluated using Bio-Plex Manager Software 6.2 (Bio-Rad Laboratories). The intra- and inter-assay coefficients of variation were lower than 10%.

### 4.7. Adenylyl Cyclase Assay

Membranes from the hippocampus were prepared as previously described [69]. Adenylyl cyclase activity was measured in membranes from the hippocampus (0.06 mg/mL) incubated with 1.5 mM ATP, 5 mM MgSO_4_, 10 mM GTP, an ATP-regenerating system, 1 mM 3-isobutyl-1-methylxanthine, 0.1 mM phenylmethylsulphonyl fluoride, 1 mg/mL bacitracin, 1 mM EDTA, and 10^−4^ M SRIF. After a 15 min incubation at 30 °C, the reaction was stopped by heating. After cooling, 0.2 mL of an alumina slurry (0.75 g/mL in Tris/HCl buffer, pH 7.4) was added, and the suspension was centrifuged. The supernatant was employed for the assay of cyclic AMP [70].

### 4.8. Cell Cultures and Treatments

#### 4.8.1. Culture of Rat Hippocampal Neurons

Cultures were performed as reported [43]. Briefly, pregnant Sprague Dawley rats were sacrificed and 18-day rat embryos collected. Hippocampi were dissected in Neurobasal medium (Gibco-Invitrogen, Madrid, Spain) containing 10% of fetal bovine serum (FBS, Gibco-Invitrogen). The cell suspension was centrifuged for 10 min at 600× *g*. The pellet was resuspended in fresh medium, and the cells were plated at a density of 5 × 10^6^ cells/dish in poly-D-lysine 100 mm Petri dishes. After 10 days of culture, the neurons were treated for 24 h with 1 μM Aβ25-35 alone or in combination with 100 μM GPE for 24 h. We measured the phosphorylated and total levels of STAT3 and IRS-1 and IDE concentrations in the lysates by a multiplexed bead immunoassay and an ELISA, respectively.

#### 4.8.2. Mixed Glial Culture

For this culture, 3–5-day-old Sprague Dawley rats were used. Briefly, the rats were sacrificed, and hippocampi were dissected by pipetting in Dulbecco’s Modified Eagle Medium (DMEM)/F12 medium (Thermo Fisher, Madrid, Spain) supplemented with 20% of FBS. Then, the cells were filtered using a 40 μm cell strainer and centrifuged for 8 min at 900× *g*. Finally, the cells were seeded in DMEM/F12 with 20% FBS at a density of 5 × 10^6^ cells/dish in 100 mm Petri dishes and cultured at 37 °C in humidified 5%CO_2_/95% air. Once confluence was achieved after 7–10 days, glial cells were treated with DMEM/F12 with 10% FBS alone (basal condition), with 1 μM Aβ25-35 alone and with 1 μM Aβ25-35 plus 100 μM GPE for 24 h. In cell lysates from glial cultures, we determined phosphorylated and total levels of STAT3 and IRS-1 and IDE content and in the extracellular culture media, we measured IFN-γ, IL-2, IL-13, and IL-17A concentrations by a multiplexed bead immunoassay.

### 4.9. Statistical Analysis

Data are summarized as mean ± SEM. The analysis of all data was carried out using one-way ANOVA followed by Bonferroni’s post hoc tests. Relationships between variables were performed by linear regression analysis. Values were considered significantly different when the *p* value was less than 0.05. Analyses were performed using Statview software (Statview 5.01, SAS Institute, Cary, NC, USA), and graphs were generated using GraphPad Prism 8 (San Diego, CA, USA) software.

## 5. Conclusions

**Figure 8:**
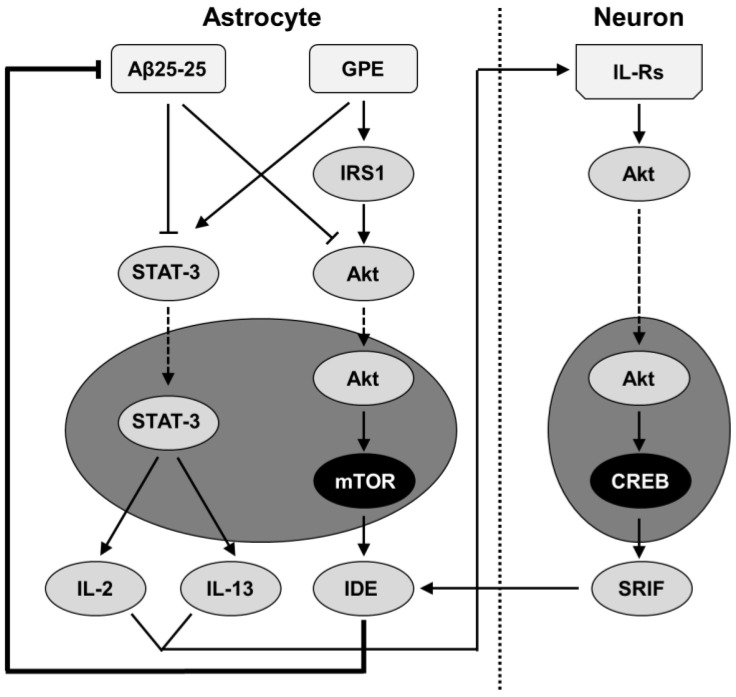
Proposed model for reduction in Aβ25-35 levels by activation of leptin- and IGF-related signaling in Aβ-treated rats after co-administration of GPE. Infusion of Aβ25-35 reduces leptin signaling, whereas GPE preserves it. The activation of STAT3 after GPE co-administration increases hippocampal levels of IL-2 and IL-13. Aβ25-35 can also block Akt signaling, and GPE counteracts Aβ effects by increasing mTOR activation and IDE synthesis. The increase in hippocampal IL levels may activate their receptors and augment Akt phosphorylation, which activates CREB and increases SRIF synthesis. These events may augment IDE levels and activity in the hippocampus, thereby reducing Aβ levels. Aβ, beta-amyloid; Akt, protein kinase B; CREB, cAMP responsive element binding protein; GPE, glycine-proline-glutamate; IDE, insulin-degrading-enzyme; IRS1, insulin receptor substrate 1; SRIF, somatostatin; STAT-3, signal transducer and activator of transcription 3. Dark ellipses indicate previously published results.

As summarized in Figure 8, our results show that GPE activates signaling pathways that modulate the inflammatory milieu. These changes may increase the levels of one of the key Aβ-degrading enzymes, with a subsequent decrease in amyloid burden, one of the major hallmarks of this neurodegenerative disease. Given the limited success in the development of therapies for AD, GPE could be a successful tool to reduce one of the main factors affecting the development of this disease and therefore represents a possible future perspective for the treatment of this disease.

## Figures and Tables

**Table 1:** Correlation between Aβ25-35 levels, SRIF inhibition of AC activity, and IDE levels with the phosphorylation of intracellular signaling targets and cytokine content in the hippocampus.

	Aβ25-35 (pg/mg)		SRIF Inhibition of AC (%)		IDE (ng/mg)	
	r	*p*	r	*p*	r	*p*
p-p38MAPK/MAPK (%)	+0.53	*	−0.59	**	−0.45	*
pSerNFκB/NFκB (%)	−0.40	NS	+0.49	*	+0.38	NS
pTyrSTAT3/STAT3 (%)	−0.74	***	+0.57	**	+0.60	**
pSerSTAT3/STAT3 (%)	−0.76	***	+0.65	**	+0.71	***
pTyrIGF-IR/mg protein	−0.63	**	+0.61	**	+0.53	*
pTyrIRS1/IRS1 (%)	−0.61	**	+0.41	NS	+0.42	NS
pSerIRS1/IRS1 (%)	+0.86	***	−0.67	**	−0.72	***
pThrAkt/Akt (%)	−0.66	**	+0.62	**	+0.55	*
IFN-γ (pg/mg)	+0.80	***	−0.60	**	−0.72	***
IL-2 (pg/mg)	−0.50	*	+0.37	NS	+0.70	***
IL-13 (pg/mg)	−0.78	***	+0.51	**	+0.69	***
IL-17A (pg/mg)	+0.60	**	−0.54	**	−0.59	**

AC, adenylate cyclase; pThrAkt, Akt phosphorylated (p) at Thr308; IFN-γ, interferon-γ; pTyrIGF-IR, insulin-like growth factor-I receptor (IGF-IR) phosphorylated (p) at Tyr1131; IL, interleukin; pTyrIRS1, insulin receptor substrate 1 (IRS1) phosphorylated at Tyr residues (pTyrIRS1), pSer636IRS1, IRS1 phosphorylated at Ser636; p-p38MAPK/MAPK, p38 mitogen-activated protein kinase (pMAPK) phosphorylated at Thr180 and Tyr182; pSerNFκB, nuclear factor kappa B (NFκB) phosphorylated at Ser536; SRIF, somatostatin; pTyrSTAT3, signal transducer and activator of transcription 3 (STAT3) phosphorylated at Tyr705; pSerSTAT3, STAT3 phosphorylated at Ser727. Correlation coefficients (*r*) and *p* values are provided for each analysis. N = 5. NS, non-significant. * *p* < 0.05, ** *p* < 0.01, *** *p* < 0.001.

## Data Availability

All relevant data are included within the manuscript.

## Abbreviations

Aβ	Amyloid-β peptide
AC	Adenylate cyclase
AD	Alzheimer´s disease
Akt	Protein kinase B
ANOVA	Analysis of variance
APP	Amyloid precursor protein
AU	Absorbance units
DMEM	Dulbecco’s modified Eagle medium
ELISA	Enzyme-linked immunosorbent assay
FBS	Fetal bovine serum
GFAP	Glial fibrillary acidic protein
GPE	Glycine-proline-glutamate
HRP	Horseradish peroxidase
IDE	Insulin-degrading enzyme
IFN-γ	Interferon-γ
IGF-I	Insulin-like growth factor I
IGF-IR	IGF-I receptor
IL	Interleukin
IRS1	Insulin receptor substrate 1
JAK2	Janus kinase 2
MFI	Median fluorescent intensity
NFκB	Nuclear factor kappa B
Ovx	Ovariectomized
p	Phosphorylated
PI3K	Phosphatidylinositol 3-kinase
PS1	Presenilin-1
p38MAPK	p38 mitogen-activated protein kinase
SOCS3	Suppressor of cytokine signaling 3
SRIF	Somatostatin
STAT3	Signal transducer and activator of transcription 3
